# Improvement of Microstructure and Mechanical Properties of a Hot-Extruded Cu-Al_2_O_3_ Alloy After Thermomechanical Treatment

**DOI:** 10.3390/ma18071606

**Published:** 2025-04-02

**Authors:** Xu Wang, Xiaoqian Pan, Pengpeng Liu, Zhu Xiao, Tao Zhou, Chunlei Gan, Juan Wang

**Affiliations:** 1 School of Materials Science and Engineering, Central South University, Changsha 410083, China; 2 NIO, Shanghai 201800, China; 3 Key Laboratory of Non-Ferrous Metal Materials Science and Engineering, Ministry of Education, Changsha 410083, China; 4 Institute of New Materials, Guangdong Academy of Sciences, Guangzhou 510650, China

**Keywords:** Cu-Al_2_O_3_ alloy, thermomechanical treatment, Orowan strengthening

## Abstract

This article presented an investigation into the microstructure evolution of a hot-extruded Cu-0.23Al_2_O_3_ alloy during thermomechanical treatment. The results demonstrated that cold rolling deformation introduced high-density dislocations into the matrix, resulting in a significant enhancement in the strength of the Cu-0.23Al_2_O_3_ alloy. Subsequent annealing at 500 for 1 h led to a reduction in dislocation density in the sample. Consequently, the strength of the sample decreased very slightly, while the elongation increased from 14% to 39%. There was little growth of the nano-scale Al_2_O_3_ particles due to their excellent thermal stability, with the average size remaining approximately 10 nm after annealing. The comprehensive properties of the Cu-0.23Al_2_O_3_ alloy were improved synchronously by thermomechanical treatment, with a tensile strength of 301 MPa and an electrical conductivity of 98.5%IACS. The calculation results of the strengthening mechanism indicated that refinement strengthening, work hardening and Orowan strengthening mainly contributed to the high strength of the Cu-0.23Al_2_O_3_ alloy.

## 1. Introduction

Pure copper or traditional high-strength and high-conductivity copper alloys, such as Cu-Cr alloys, exhibit excellent electrical and thermal conductivity. However, their relatively low strength at elevated temperatures and unsatisfactory high-temperature softening behavior gradually limit their use in many thermal management scenarios [1,2,3]. In recent years, oxide dispersion-strengthened (ODS) copper alloys have received extensive attention. ODS Cu alloys are a kind of composite material with copper as the matrix and ceramic particles as the reinforcement phase, including Al_2_O_3_, Y_2_O_3_, ZrO_2_ and TiO_2_ [4,5,6,7,8]. These ceramic particles are characterized by high hardness and high thermal stability. The ODS alloys exhibit excellent comprehensive properties, including high electrical conductivity, thermal conductivity and resistance to high-temperature softening. It is particularly noteworthy that the Cu-Al_2_O_3_ alloys prepared by the internal oxidation method have a wide range of applications in domains such as electronic packaging, vehicle welding and weapons [9,10,11,12].

Generally speaking, the preparation process of Cu-Al_2_O_3_ alloys is powder mixing, cold pressing, internal oxidation/reduction, hot extrusion, annealing and so on. In order to improve the plastic deformation ability of the Cu-Al_2_O_3_ alloy without reducing the strength too much, the alloy can be hot rolled after hot extrusion. Dong et al. [13] carried out hot rolling deformation on the Cu-5 vol%Al_2_O_3_ alloy after hot extrusion. The spatial distribution of nano-scale Al_2_O_3_ particles in the Cu matrix was improved, and the elongation of the alloy increased from 6% to 12% without reducing the strength of the alloy. This was due to the interaction between nano-scale Al_2_O_3_ particles, geometrically necessary dislocations and the coarse grains containing movable dislocations. It is also an effective way to add other elements to the ODS Cu alloys to promote the precipitation of other strengthening phases except Al_2_O_3_. Song et al. [9] prepared an Al_2_O_3_/Cu-0.15Cr alloy with Cu-0.3Al alloy powder, Cu-0.3Al alloy powder and Cu_2_O powder as raw materials. Solid solution, cold rolling and aging were carried out on the alloy after hot extrusion. The mechanical properties of 1.4% Al_2_O_3_/Cu-0.3Cr were improved jointly, with a hardness of 150 HV, a tensile strength of 510 MPa and an elongation of 15%. Meanwhile, the electrical conductivity was above 85%IACS. Al_2_O_3_ particles slowed down the recrystallization process and promoted the precipitation of Cr by pinning dislocation. Because ODS copper alloys contain a large number of dispersed particles, their deformation mechanism and microstructure evolution also show unique characteristics. Liu et al. [14] conducted split Hopkinson pressure bar (SHPB) tests to study the plastic deformation and dynamic recrystallization mechanism of Cu-0.83 wt%Al_2_O_3_ alloy at high strain rates. The results showed that the alloy exhibited higher strain rate sensitivity and higher flow stress under the conditions of a high strain rate. While the strain increased, the texture of the alloy changed from a Gauss texture and a Cube texture to a R-Cube texture. However, the volume fraction of the ceramic phase in most Cu-Al_2_O_3_ alloys is high, which inevitably leads to a decrease in electrical conductivity. It is difficult to meet the electrical performance requirements in specific application scenarios. Therefore, it is necessary to study ODS copper with low Al_2_O_3_ content. Furthermore, considering the complex shape and high dimensional accuracy of some specific products (such as electrode caps), how to improve the plastic deformation ability of Cu-Al_2_O_3_ alloy also needs more research.

In this paper, a Cu-Al_2_O_3_ alloy with low Al_2_O_3_ content of 0.23 vol% was fabricated by the traditional internal oxidation method. A combined process route of hot extrusion, cold rolling and annealing was designed to improve the plastic deformation ability. The property variation and the effects of Al_2_O_3_ particles on microstructure change during deformation were characterized. Finally, a Cu-0.23 vol%Al_2_O_3_ alloy with excellent comprehensive properties was prepared. This paper provided theoretical guidance for the preparation and processing of ODS copper alloys with low Al_2_O_3_ content.

## 2. Experimental Procedures

The raw materials for preparing the Cu-Al_2_O_3_ alloy were Cu-0.05 wt%Al powder (prepared by vacuum melting and high-purity nitrogen atomization) and Cu_2_O powder. The main process included mixing powders, cold isostatic pressing, internal oxidation sintering, reduction and hot extruding. The raw material powders were weighed according to a specific ratio and placed in a planetary ball mill (QM-3SP4, Nanjing Laibu Technology Industrial Co., Ltd., Nanjing, China) for mixing at a rotating speed of 100 rpm for 4 h. The mixed powder was filled into a soft rubber sleeve and pressed at 180–200 MPa using a cold isostatic pressing machine. The compacts underwent internal oxidation sintering in a tube furnace under the protection of argon atmosphere at 900 °C for 4 h. Similarly, reduction process was conducted under Ar-10 vol%H_2_ atmosphere at 920 °C for 4 h to remove redundant Cu_2_O. Then, the compacts were hot-extruded at 930 °C with an extrusion ratio of 20:1. The hot extrusion was carried out on a 500t vertical hydraulic press (YQ32-500, Tengzhou nanduan numerical control machine tool Co., Ltd., Tengzhou, China). Before extrusion, the mold was heated to about 450 °C, and graphite oil was used for lubrication during extrusion. Cold rolling deformation was carried out by a double roll mill (Φ420 × 350, Yunnan Metallurgy Kunming Heavy Industry Co., Ltd., Kunming, China), and subsequent annealing was performed in tube furnace (KSL-1200X, Hefei Kejing Materials Technology Co., Ltd., Hefei, China) at 500 °C for 1 h. The samples after hot extrusion, cold rolling and annealing were designated as HE, HE-CR and HE-CR-500 °C/1 h, respectively.

The actual chemical composition of the alloy was measured by a SPECTROBLUE inductively coupled plasma–optical emission spectrometer (ICP-OES, SPECTRO BLUE, SPECTRO Analytical Instruments, Kleve, Germany). The result is Cu-0.1 wt%Al_2_O_3_ and can be converted to Cu-0.23 vol%Al_2_O_3_ according to the density of Cu and Al_2_O_3_. The Vicker hardness of the samples was measured using a digital microhardness tester (HV-1000, Laizhou Huayin test Instrument Co., Ltd., Laizhou, China) equipped with a diamond indenter. The applied load was 300 g, and the hold time was 15s. Electrical conductivity was tested using an FD-102 (Xiamen First Electronic Technology Co., Ltd., Xiamen, China) eddy current conductivity meter with frequency of 60 kHz. Each sample was tested five times, and the average value was chosen. Mechanical test was conducted on MTS-810 test machine (MTS Systems Co., Ltd., Eden Prairie, MN, USA) using dog-bone-shaped specimens with strain rate of 0.001 s^−1^, and three samples were tested for each state. The length, width and thickness of dog-bone-shaped specimens were 20 mm, 8 mm and 1.5 mm, respectively. Electron backscattered diffraction (EBSD, Oxford C-nano, Oxford Instruments, Abingdon, UK) and transmission electron microscope (TEM, Talos F200X, Thermo Fisher Scientific Inc., Waltham, MA, USA) were applied to observe microstructure evolution and phase distribution. The acceleration voltage and scan step during EBSD characterization were 20 kV and 0.15 μm, and the acceleration voltage during TEM characterization was 200 kV.

## 3. Results

### 3.1. Microstructure of Cu-0.23Al_2_O_3_ Alloy

**Figure 1:**
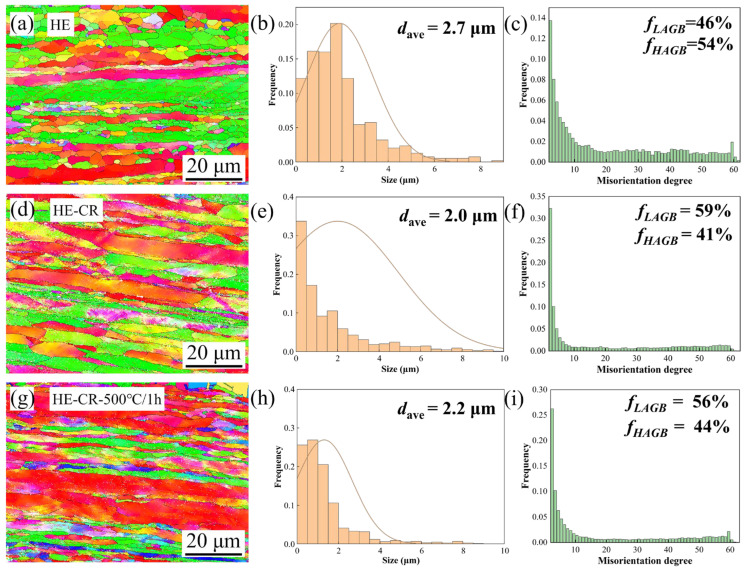
EBSD characterization results of Cu-0.23Al_2_O_3_ alloy in different states. (**a**,**d**,**g**) IPF coloring images of HE, HE-CR and HE-CR-500 °C/1 h samples; (**b**,**e**,**h**) grain size distribution in the samples; (**c**,**f**,**i**) grain boundary angle distribution in the samples.

Figure 1 shows the EBSD characterization results of the Cu-0.23Al_2_O_3_ alloy in different states. Figure 1a,d,g are inverse pole figure (IPF) coloring images of the HE, HE-CR and HE-CR-500 °C/1 h samples. Black and red lines represent low-angle grain boundaries (2°–10°) and high-angle grain boundaries (>10°), respectively. It can be seen that most of the grains are elongated along the extrusion direction in the HE sample (Figure 1a). There are a large number of low-angle grain boundaries inside the elongated grains, which divide the grains into subgrains. Due to the occurrence of dynamic recrystallization, some equiaxed grains are observed. There are few low-angle grain boundaries in the sample. After cold rolling, the previously recrystallized equiaxed grains transform into a fiber structure in the HE-CR sample (Figure 1d). The color gradient in the fiber structure can be clearly seen, indicating the formation of deformation bands due to dislocation slipping in these grains. The grain morphology in the HE-CR-500 °C/1 h sample is similar to that in the HE-CR sample. Figure 1b,e,h represent grain size distribution in HE, HE-CR and HE-CR-500 °C/1 h samples. In the HE sample, the grain size is mainly concentrated around 2 μm. After cold rolling and annealing, the size of most grains decreases slightly below 1 μm. The average grain size of the three samples is similar, which were 2.7 μm, 2.0 μm and 2.2 μm, respectively. Figure 1c,f,i show the distribution of grain boundary angles in the HE, HE-CR and HE-CR-500 °C/1 h samples. In the HE sample, due to the coexistence of subgrains and recrystallized grains, the fractions of low-angle grain boundaries and high-angle grain boundaries are close (46% and 54%). After cold rolling, high-density dislocations are introduced to the sample. The content of low-angle grain boundaries increases, and the content of high-angle grain boundaries slightly decreases. The content of low-angle grain boundaries and high-angle grain boundaries in HE-CR are 59% and 41%, respectively. While in the HE-CR-500 °C/1 h sample, they are 56% and 44%, respectively. It can be safely suspected that the transformation from low-angle grain boundaries to high-angle grain boundaries is associated with recrystallization [5]. Considering that the increase in the fraction of high-angle grain boundaries after annealing is not significant, it can be determined that recovery mainly occurs during annealing. The response process hardly involves the generation and migration of high-angle grain boundaries [15].

**Figure 2:**
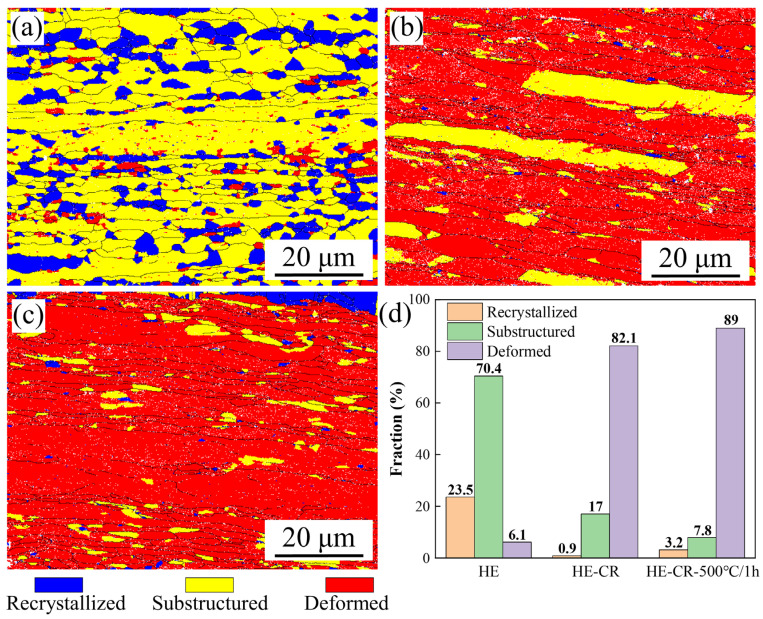
Distribution of recrystallization, substructure and deformed structure in the samples. (**a**) HE; (**b**) HE-CR; (**c**) HE-CR-500 °C/1 h; (**d**) specific fraction of different structures in the samples.

Figure 2a–c show the distribution of recrystallization, substructures and deformed structures in Cu-0.23Al_2_O_3_ alloys under different states, and the specific fractions of different structures were listed in Figure 2d. In the HE sample, the fraction of recrystallization, substructures and deformed structures are 23.5%, 70.4% and 6.1%, respectively. Due to the occurrence of dynamic recovery and dynamic recrystallization during hot extrusion, only a few deformed structures are detected with sporadic distribution. The recrystallized structures were mostly equiaxed and evenly distributed in the sample. In the HE-CR and HE-CR-500 °C/1 h samples, the deformed structures are dominant with their fraction of 82.1% and 89%, respectively. Only a small amount of recrystallized structures and substructures remain.

**Figure 3:**
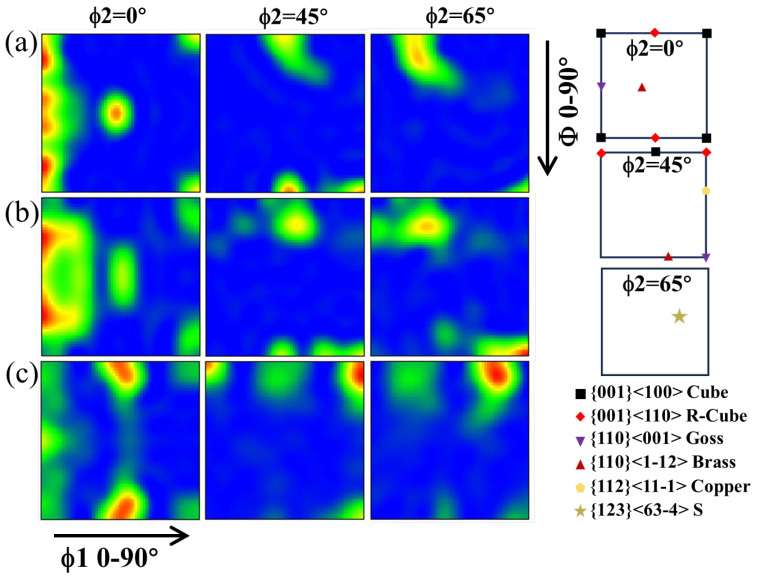
ODF sections of the samples of different states. (**a**) HE; (**b**) HE-CR; (**c**) HE-CR-500 °C/1 h.

Figure 3a–c show the orientation distribution function (ODF) sections (ϕ2 = 0°, 45°, 65°) of the HE, HE-CR and HE-CR-500 °C/1 h samples. The location of the typical texture in FCC metals is shown on the right. In the EH sample, the Cube texture, Goss texture and Brass texture are dominant. After cold rolling, the intensity of the above texture gradually decreases, and a weak S texture is generated. The texture types in the HE-CR sample are in accord with the results in ref. [16]. Annealing results in the domination of the R-Cube texture while a small amount of the Goss texture and the Cube texture still remain.

**Figure 4:**
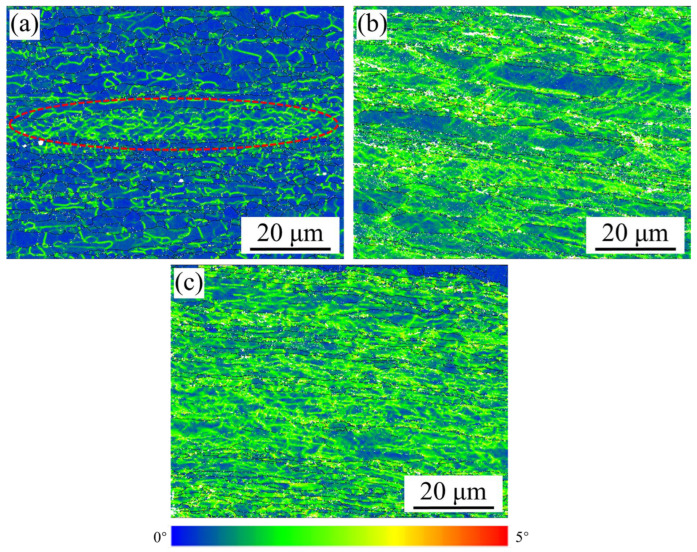
KAM maps of the samples of different states. (**a**) HE; (**b**) HE-CR; (**c**) HE-CR-500 °C/1 h.

Figure 4a–c display the kernel average misorientation (KAM) maps of the HE, HE-CR and HE-CR-500 °C/1 h samples, reflecting the degree of lattice distortion. As shown in Figure 4a, most regions have a lower KAM value, indicating lower lattice distortion in the HE sample. However, the KAM value in the dotted lines marked region is higher than that in other areas. Referring to Figure 1a and Figure 2a, the marked region responds to the substructure region containing a large number of low-angle grain boundaries. In the HE-CR and HE-CR-500 °C/1 h samples, the KAM value distribution is uniform. Due to the application of cold deformation, the KAM value in them is higher than that in the EH sample. Based on the KAM value, the dislocation density of the samples could be calculated by the following equation [17]:

(1) $$\rho =\frac{2KAM}{\mu b}$$

where μ is the step size of scanning of EBSD test, and *b* is the Burgers Vector of copper. Finally, the dislocation densities of the three samples are calculated to be 1 × 10^12^ m^−2^, 1.9 × 10^14^ m^−2^ and 9 × 10^13^ m^−2^, respectively. Cold rolling deformation doubles the dislocation density in the sample, while annealing results in a decrease in dislocation density. Recovery partially returns the microstructure to its initial state through the thermally activated annihilation and rearrangement of dislocations [18].

**Figure 5:**
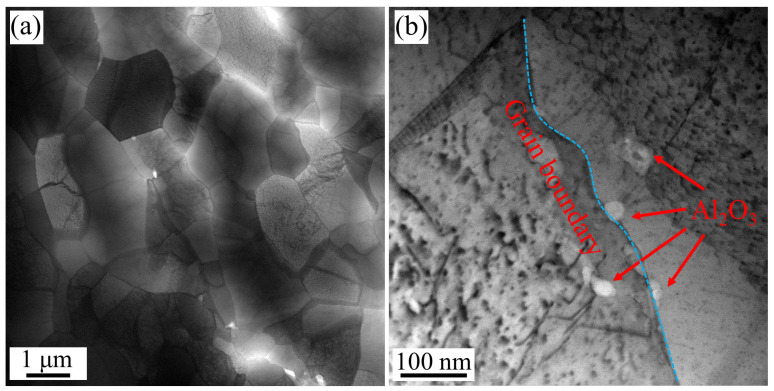
TEM images of HE sample. (**a**) Equiaxed recrystallized; (**b**) Al_2_O_3_ particles at grain boundaries and dislocations.

Figure 5 shows the TEM images of the HE sample. Equiaxed recrystallized grains are observed in Figure 5a. Most of the grains have undergone perfect recrystallization, and the interior of the grains is clean. During hot deformation, dislocations undergo slipping, entanglement and annihilation. Polygonization, cell regularization and subgrain merging occur subsequently. Ultimately, recrystallized grains are generated. There are still several dislocations remaining inside some grains. The rate of dislocation proliferation in these grains is greater than that of dislocation annihilation, resulting in the generation of substructure. Several Al_2_O_3_ particles distribute at grain boundaries, and dislocations can be observed in the high-magnification image (Figure 5b). Al_2_O_3_ particle is a hard phase that can effectively pin the migration of grain boundaries and dislocation slipping. This is the reason why Cu-Al_2_O_3_ alloys have excellent softening resistance.

**Figure 6:**
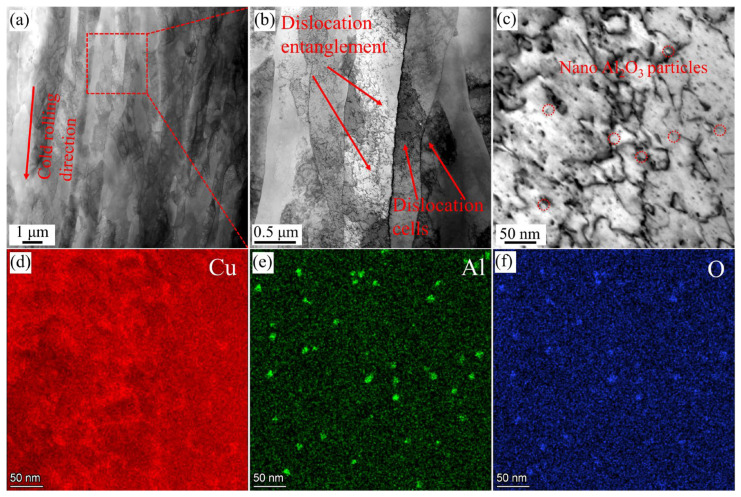
TEM images of HE-CR sample. (**a**) Biber structures; (**b**) various dislocation configurations; (**c**) nano-sized Al_2_O_3_ particles pinning dislocation lines; (**d**–**f**) EDS mapping images of Cu, Al and O elements corresponding to Figure 7c.

Figure 6 shows the TEM images of the HE-CR sample. Grains are elongated to form fiber structures parallel to the rolling direction, and high-density dislocations are generated simultaneously (Figure 6a). It can be seen that there are various dislocation configurations, such as dislocation entanglement, dislocation cells and dislocation walls inside the fiber structures, as shown in Figure 6b. Figure 6c shows the pinning of nano-sized Al_2_O_3_ particles (marked by circles) to dislocation lines. Figure 6d–f are the EDS mapping images corresponding to Figure 6c. Nano-Al_2_O_3_ particles are uniformly distributed in the matrix, with most of them having a size of around 10 nm.

**Figure 7:**
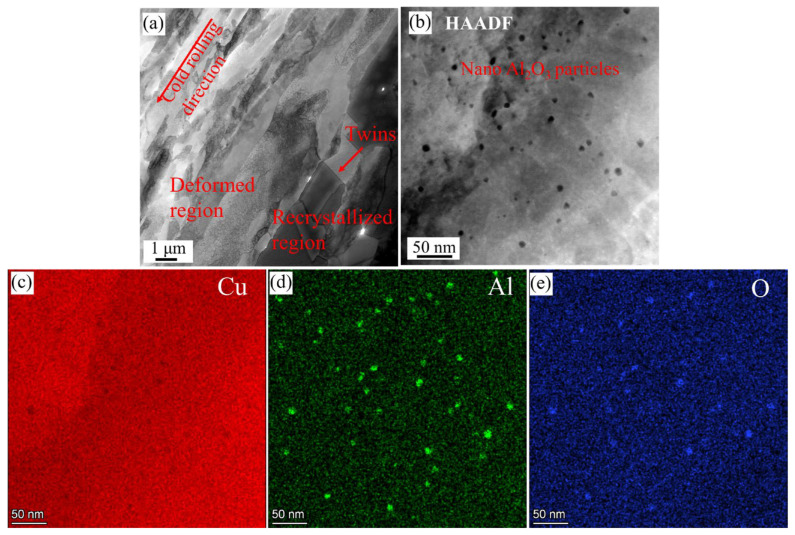
TEM images of HE-CR-500 °C/1 h sample. (**a**) Deformation region and recrystallized region; (**b**) nano-sized Al_2_O_3_ particles; (**c**–**e**) EDS mapping images of Cu, Al and O elements corresponding to Figure 8b.

Figure 7 shows the TEM images of the HE-CR-500 °C/1 h sample. During the annealing process, recrystallization happens. There are few dislocations in the recrystallized region, the grain boundaries are relatively straight and annealing twins can be observed (Figure 7a). The characteristics of the deformation region are similar to those after cold rolling, but the dislocation density slightly decreases, which is consistent with the EBSD results. Figure 7b–e are high-magnification HAADF images of the recrystallized region and corresponding EDS mapping images. The size of nano-Al_2_O_3_ particles remains almost unchanged before and after annealing.

### 3.2. Properties of Cu-0.23Al_2_O_3_ Alloy

**Figure 8:**
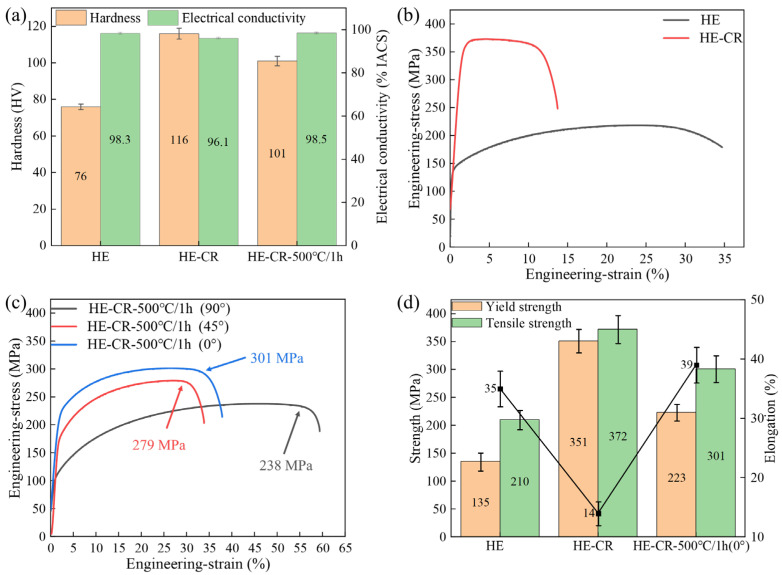
(**a**) Hardness and electrical conductivity of the samples; (**b**) engineering stress–strain curves of HE and HE-CR samples; (**c**) engineering stress–strain curves of HE-CR-500 °C/1 h samples with various orientations; (**d**) mechanical properties of the samples.

Figure 8a shows the hardness and electrical conductivity of the samples. Due to dynamic recovery and recrystallization during the hot deformation, the hardness of the HE sample is only 76 HV, and its electrical conductivity is as high as 98.3%IACS. This reflects that during the internal oxidation, Al fully reacts to form Al_2_O_3_ particles, and only trace amounts of Al dissolve in the matrix. After cold deformation, the hardness of the sample increases to 116 HV due to work hardening. At the same time, lattice distortion results in scattering during electron transport, and the electrical conductivity of the sample decreases to 96.1%IACS. The hardness and electrical conductivity of the HE-CR-500 °C/1 h sample are 101 HV and 98.5%IACS, respectively. Figure 8b,c show the engineering stress–strain curves of the samples. It is worth mentioning that the anisotropy of the HE-CR-500 °C/1 h samples are considered. The tensile samples are taken at different angles from the rolling direction (0°, 45° and 90°, Figure 8c). There is obvious anisotropy on the HE-CR-500 °C/1 h samples that when the tensile direction is parallel to the rolling direction, the sample exhibits the highest tensile strength of 301 MPa. While the tensile strength is only 238 MPa for the sample tested perpendicular to the rolling direction. Hence, in the process of application, attention should be paid to its anisotropy. Figure 8d shows the specific values of the mechanical properties of the samples. The tensile strength of the samples in the three states are 210 MPa, 372 MPa and 301 MPa, respectively. Although annealing reduces the strength of the alloy, it provided space for dislocation propagation during subsequent deformation. Therefore, the elongation of the HE-CR-500 °C/1 h sample significantly increases to 39% after annealing. A higher elongation indicated that the sample has excellent ductility, which is beneficial for the product formability of the alloy. Overall, after the combination of hot deformation, cold deformation and annealing, the strength of the sample meets the performance requirements of the electrode cap for spot welding and also possesses good plastic formability.

## 4. Discussion

### 4.1. Effect of Al_2_O_3_ Particles on Recrystallization

Only slight recrystallization occurs in the HE-CR-500 °C/1 h sample, with a fraction of 3.2% (Figure 2d). However, for pure copper, the sample with 50% rolling reduction undergoes complete recrystallization after annealing at 400 °C for 1 h [19]. The rolled Cu-0.23Al_2_O_3_ alloy and pure copper exhibit different recrystallization behaviors during annealing. The former has a strong resistance to recrystallization, while the latter can undergo significant recrystallization at even lower temperatures. The dispersed fine Al_2_O_3_ particles in Cu-0.23Al_2_O_3_ alloy play an important role. Zener pinning refers to the retarding force of small particles to the movement of grain boundaries [20], which can be expressed as

(2) $$P_{Z}=\frac{3f_{v}\gamma_{AB}}{2r}$$

$f_{v}$ is the volume fraction of Al_2_O_3_ particles. $\gamma_{AB}$ is the boundary tension, and *r* is the particle radius. Al_2_O_3_ particles maintain nano-scale during the annealing, which ensures a high $P_{Z}$ value. Therefore, the movement of grain boundaries is restricted. Meanwhile, the critical size for recrystallization nucleation (*R*) is inversely proportional to $P_{Z}$ [15]:

(3) $$R=\frac{\delta \gamma_{AB}}{{P_{D}-P}_{Z}}$$

where δ is a constant related to grain shape, and $P_{D}$ is the driving force for recrystallization. In summary, the nucleation and growth of recrystallization are inhibited.

### 4.2. Strengthening Mechanism

**Figure 9:**
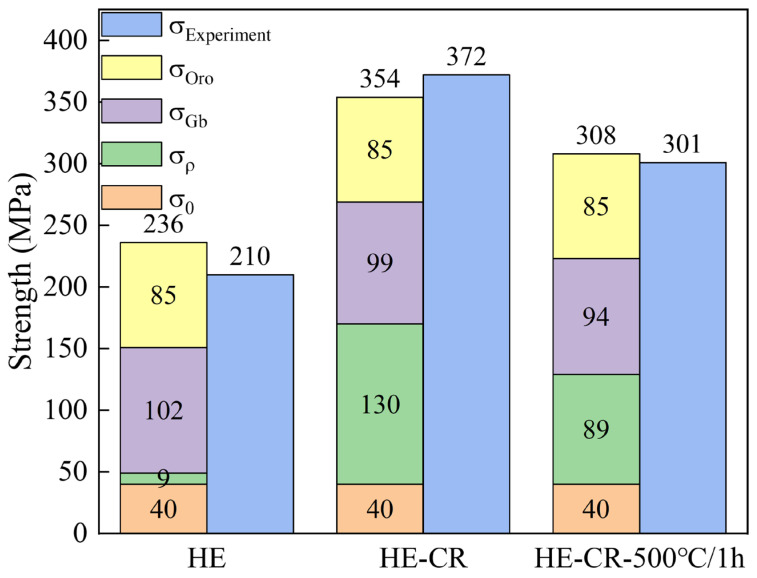
Calculation results of strengthening mechanism.

The strength of the samples in this article is higher than that of annealed pure copper, with the reinforcement phase Al_2_O_3_ playing an important role. Generally speaking, the strengthening mechanisms in copper alloys mainly include solid solution strengthening, refinement strengthening, Orowan strengthening and work hardening. For the Cu-0.23Al_2_O_3_ alloy, due to the sufficient oxidation of Al and reduction of excess Cu_2_O, it can be considered that there are few solute atoms in the copper matrix. Hence, solid solution strengthening can be ignored. The contribution of various strengthening mechanisms can be calculated using the following equation [21]:

(4) $$\sigma =\sigma_{0}+\sigma_{\rho }+\sigma_{Gb}+\sigma_{Oro}$$

where $\sigma_{0}$ is the intrinsic strength of pure copper. The work hardening caused by dislocation accumulation can be estimated using Taylor’s formula [22]:

(5) $$\sigma_{\rho }=\alpha GMb\sqrt{\rho }$$

where α is constant, G is the shear modulus of copper and M is the Taylor factor. Refinement strengthening comes from the hindering effect of grain boundaries on dislocation slipping, which can be expressed using the Hall–Petch formula [23]:

(6) $$\sigma_{Gb}=k\sqrt{\frac{1}{d}}$$

where *k* is constant, and *d* is the average grain size. When the dislocation lines encounter non deformable Al_2_O_3_ particles, they cannot cut through the particles and only bypass to form dislocation loops around them. This increases the resistance of the material to continue deformation and causes Orowan strengthening. Orowan strengthening can be calculated by [24]

(7) $${\Delta \sigma }_{Oro}=\frac{0.81MGb\ln\left(d_{p}/b\right)}{\pi {\left(1-v\right)}^{\frac{1}{2}}(d_{p}\sqrt{\frac{3\pi }{2f_{v}}}-2d_{p})}$$

where v is Poisson’s ratio, and $d_{p}$ is the size of Al_2_O_3_ particles. According to the SEM and TEM characterization results above, Al_2_O_3_ particles in the alloy have dual scales. One is coarse submicron-sized, and the other is nano-sized. For Orowan strengthening, the effect of nano-scale particles is much higher than that of submicron ones. Therefore, the effects of submicron particles are not considered here. Table 1 shows the parameters used in the calculation process. Figure 9 shows the calculation results of each strengthening mechanism and the comparison between the calculated values and experimental values. The calculated results are consistent with the experimental values. It can be concluded that refinement strengthening and Orowan strengthening from Al_2_O_3_ particles mainly contribute to the strength increment of the HE sample. When dislocations are introduced through cold deformation, work hardening becomes dominant. After annealing, the effect of work hardening weakens. The contributions of work hardening, refinement strengthening and Orowan strengthening are almost equivalent. In the samples, the Orowan strengthening remains consistent thanks to the high thermal stability of Al_2_O_3_ particles, which do not coarsen or dissolve during cold deformation or annealing.

### 4.3. Properties Comparison

**Figure 10:**
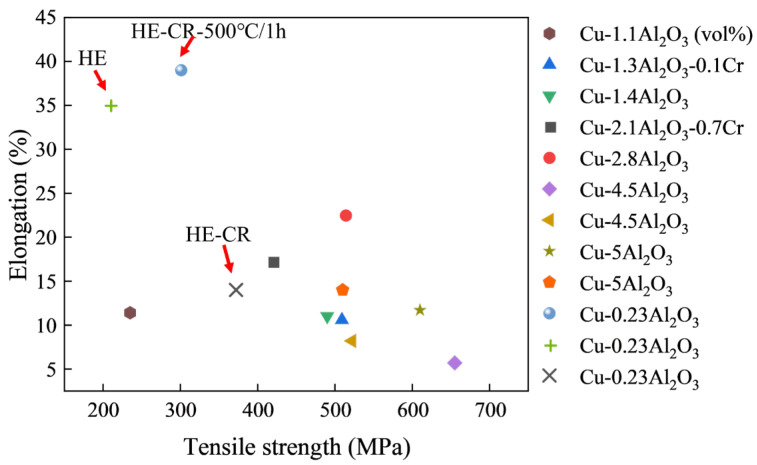
The comparison of elongation and tensile strength between the Cu-Al_2_O_3_ alloys in this article and other recently published studies.

Figure 10 shows the comparison of elongation and tensile strength between the Cu-Al_2_O_3_ alloys in this article and other recently published studies [9,13,21,25,26,27,28]. It is obvious that there is a contradictory relationship between the strength and the elongation of the alloy. Alloys with higher strength tend to have lower elongation. In addition, the properties of the alloys are closely related to the volume fraction of the Al_2_O_3_ particles. For example, for the Cu-4.5Al_2_O_3_ alloy in Ref [26], the contribution of Orowan strengthening is calculated to be 244 MPa, which is much higher than the result in this article (85 MPa). The size of Al_2_O_3_ particles is also important to the Orowan strengthening. In the Cu-1.4Al_2_O_3_ [9] and Cu-2.8Al_2_O_3_ [21] alloy, where the volume fractions of Al_2_O_3_ particles are different, the Orowan strengthening is calculated to be about 200 MPa. It is the particle size difference that results in the similar Orowan strengthening effect, which is 5 nm in the Cu-1.4Al_2_O_3_ alloy and 8 nm in the Cu-2.8Al_2_O_3_ alloy. In our work, the average size of Al_2_O_3_ particles is about 10 nm. The nano-scale particles also bring considerable Orowan strengthening, although there is a low volume fraction of 0.23%. In general, the HE-CR-500 °C/1 h sample has good comprehensive properties, especially the elongation attributed to the appropriate annealing treatment.

## 5. Conclusions

1. The Cu-0.23Al_2_O_3_ alloy achieves excellent comprehensive properties after being processed by thermomechanical treatment. The hardness, yield strength, tensile strength and elongation of the alloy in its final state are 101 HV, 223 MPa, 301 MPa and 39%. Because of the low content of Al_2_O_3_ in the alloy, the alloy has a high electrical conductivity of 98.5%IACS.
2. The annealing treatment decreases the dislocation density in the sample and the strength, while the elongation is improved significantly. The good ductility ensures that the plastic formability of the material can meet the requirement of manufacturing.
3. Refinement strengthening, Orowan strengthening and work hardening contribute to the increment of strength of the annealed Cu-0.23Al_2_O_3_ alloy.

## Figures and Tables

**Table 1:** The parameters used in the calculation process.

Parameter	Value
*G*	46 GPa
α	0.26
M	3.06
b	0.256 nm
v	0.34
$k_{y}$	0.14 MPa⋅m^1/2^

## Data Availability

The original contributions presented in this study are included in the article. Further inquiries can be directed to the corresponding author.
